# Custom Multiphoton/Raman Microscopy Setup for Imaging and Characterization of Biological Samples

**DOI:** 10.3390/mps2020051

**Published:** 2019-06-20

**Authors:** Marco Marchetti, Enrico Baria, Riccardo Cicchi, Francesco Saverio Pavone

**Affiliations:** 1 Department of Physics, University of Florence, 50019 Sesto Fiorentino, Italy; francesco.pavone@unifi.it; 2 National Institute of Optics, National Research Council (CNR-INO), 50019 Sesto Fiorentino, Italy; riccardo.cicchi@ino.cnr.it; 3 European Laboratory for Non-linear Spectroscopy (LENS), 50019 Sesto Fiorentino, Italy

**Keywords:** microscopy, multi-photon processes, Raman, Imaging systems, tissue characterization

## Abstract

Modern optics offers several label-free microscopic and spectroscopic solutions which are useful for both imaging and pathological assessments of biological tissues. The possibility to obtain similar morphological and biochemical information with fast and label-free techniques is highly desirable, but no single optical modality is capable of obtaining all of the information provided by histological and immunohistochemical analyses. Integrated multimodal imaging offers the possibility of integrating morphological with functional-chemical information in a label-free modality, complementing the simple observation with multiple specific contrast mechanisms. Here, we developed a custom laser-scanning microscopic platform that combines confocal Raman spectroscopy with multimodal non-linear imaging, including Coherent Anti-Stokes Raman Scattering, Second-Harmonic Generation, Two-Photon Excited Fluorescence, and Fluorescence Lifetime Imaging Microscopy. The experimental apparatus is capable of high-resolution morphological imaging of the specimen, while also providing specific information about molecular organization, functional behavior, and molecular fingerprint. The system was successfully tested in the analysis of ex vivo tissues affected by urothelial carcinoma and by atherosclerosis, allowing us to multimodally characterize of the investigated specimen. Our results show a proof-of-principle demonstrating the potential of the presented multimodal approach, which could serve in a wide range of biological and biomedical applications.

## 1. Introduction

Tissue diagnostics is usually performed by pathologists through microscopic analysis of a processed, sliced and stained tissue biopsy which has been surgically removed from the suspicious area. Despite recent improvements in the techniques used in histopathology, there is a rising need for efficient, accurate and less complex staining procedures [1]. The largely used hematoxylin and eosin (H&E) staining makes morphological analysis possible, thanks to its capability of highlighting the fine structures of cells and tissues [2]. A more detailed analysis requires the use of molecule-specific labels and an immunohistochemical approach [3], which is expensive and time-consuming without being operator-independent, thus prone to sampling errors and artifacts.

Several microscopic and spectroscopic label-free modalities have been developed over the past years, offering a wide range of techniques that can support standard histopathological methods. Among these, Multi-Photon Microscopy (MPM) [4,5,6], the analysis of inelastic scattering [7,8], Optical Coherence Tomography (OCT) [9] and others have been widely used in biomedical applications.

Among MPM techniques, Two-Photon Excited Fluorescence (TPEF) microscopy, since its invention in 1990, has undergone an impressive growth in both the biological and biomedical fields [10,11]. TPEF intrinsically offers several advantages with respect to both wide-field and confocal microscopy such as optical sectioning capability, reduced photo-damage/photo-toxicity, reduced scattering and high penetration depth in biological tissues, thanks to the use of NIR laser wavelengths. Moreover, since cells and extracellular matrix intrinsically contain a variety of fluorescent molecules, biological tissues can be imaged by TPEF microscopy without any exogenous label [12,13]. The light emitted by the specimen can be detected with a PMT in order to obtain a fast imaging of the sample under analysis, or can be temporally or spectrally resolved, respectively realizing Fluorescence Lifetime Imaging Microscopy (FLIM) or Spectral Lifetime Imaging Microscopy (SLIM). These analyses can be applied to study protein localization, fluorophores relative abundance or their molecular environment, revealing in this way functional information [14,15].

Using the same laser source, Second Harmonic Generation (SHG) microscopy can be implemented in the same setup in order to obtain additional morphological information [16]. In fact, SHG microscopy is able to selectively image non-centrosymmetric molecular structures—such as collagen fibers and cholesterol crystals [17,18]—which are relatively abundant in most biological tissues. SHG has the same intrinsic advantages of TPEF, inherent to non-linearity; moreover, both signals can be generated at the same time and co-registered by separating them simply using an optical filter. Finally, the optical coherence of the SHG phenomenon allows obtaining structural information about second-order nonlinear susceptibility tensor by measuring the behavior of the SHG signal as a function of the polarization direction of the excitation light (P-SHG) [19,20].

Raman spectroscopy is another label-free tool for inspecting the intrinsic molecular content of biological tissues [21,22]. This technique is based on the inelastic scattering of light due to the interaction between laser photons and molecular vibrational levels, resulting in energy shifts ranging from 100 to 3500 cm^−1^. The spectral analysis of the inelastically scattered light provides high detailed information on the molecular content of the sample; moreover, the use of NIR excitation wavelengths allows high penetration depth into biological tissues. Unlike SHG and TPEF, the Raman effect is a linear process, hence it can be generated by a continuous-wave laser. While this feature makes it possible to use cheaper excitation sources, it also affects the amplitude of the signal, thereby requiring longer acquisition times, and denies the possibility of having intrinsic optical sectioning.

The same molecular transitions between vibrational levels, observed with Raman spectroscopy, can be used for fast multi-photon imaging through Coherent Anti-Stokes Raman Scattering (CARS [23]). This technique needs two synchronized pulsed lasers to induce the stimulated transition and generate a signal which is orders of magnitude higher than the spontaneous process. Hence, CARS provide fast and label-free 3D imaging [24] based on the intensity of specific Raman bands; also, being a coherent phenomenon, it can be used for polarization-resolved measurements (P-CARS) [25].

Each of the aforementioned technique presents important limitations and none of them alone is able to provide the same information provided by histological and immunohistochemical analysis. P-SHG, P-CARS, FLIM or SLIM would require the acquisition of a very large number of images, taking a huge amount of time and exposing the examined sample to dangerous levels of radiation. Raman microscopy is even slower, since differences in probability and in the optical configuration of this technique severely affect the amplitude of its signal, leading to significantly higher amount of time needed to reach a comparable spatial resolution. Typical acquisition times for Raman maps can be in the order of ~1 s per point (or longer), resulting in image acquisition times in the order of hours or days. On the other hand, combining different imaging techniques could overcome these issues, but at the same time it can be challenging due to the optical and hardware requirements for different imaging systems. For these reasons, multimodal microscopy has an enormous potential for improving tissue diagnostics and being a valuable alternative to standard histopathological procedures. The integration of these techniques is the key to reaching their full potential [26,27,28], realizing fast and high-resolution 3D imaging with MPM, and integrating the images collected with morpho-chemical and functional information in a label-free, spatially targeted approach.

In this paper, we are presenting a multimodal unique optical imaging system with the capability of providing comprehensive structural, functional and molecular information of tissues in micrometer scale through the combination of all the above-mentioned optical techniques. In the manuscript, we first describe the opto-mechanical and electronic components of our microscope, characterizing its spatial and temporal resolutions, its spectroscopic capabilities, and reporting on its application in the study of both tumor and non-tumor diseases. The obtained results show the great potential of the presented multimodal approach, which could be employed for digital pathology purposes as a complementary technique to standard histological and immunohistochemical methods.

## 2. Materials and Methods

The experimental setup is composed of three parts: the opto-mechanical system for beam conditioning and scanning, the detection system, the electronics for control and acquisition. To test the microscope performances, we used gold nanoparticles and SHG emission from collagen fibers to measure its spatial and temporal resolution respectively. Then, we used the microscope to evaluate its diagnostic capabilities on two types of human tissues which were representative of both tumor and non-tumor pathology: atherosclerotic plaques and bladder tissues affected by urothelial carcinoma (UC).

### 2.1. The Opto-Mechanical System

**Figure 1:**
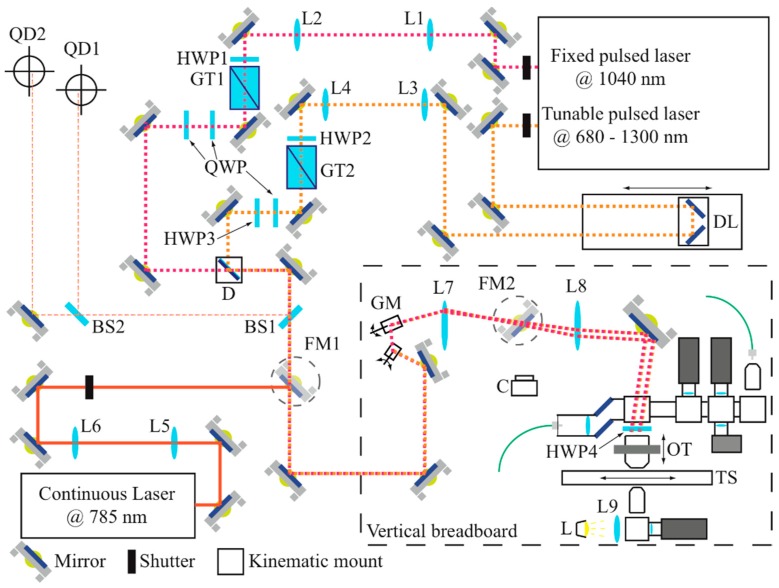
Schematic view of the opto-mechanical system. L1–L2 lenses of the tunable source telescope, L3–L4 lenses of the fixed source telescope, L5–L6 lenses of the continuous-wave laser telescope, L7 scan lens, L8 tube lens, L9 white light lens, HWP half wave plate, QWP quarter wave plate, GT Glan-Taylor polarizer, FM flipping mirrors, BS1 90/10 beam splitter, BS2 50/50 beam splitter, QD quadrant detectors, GM galvanometric mirrors, C camera, OT objective translator, TS xy-translation stage, L white light lamp.

The opto-mechanical system is schematized in Figure 1.

The laser source for multiphoton imaging is a Chameleon Discovery (Coherent, Santa Clara, CA, USA), an Yb-based pulsed laser at 80 MHz rate with two synchronous outputs: the principal is tunable from 680 to 1300 nm with pulses of about 100 fs, the auxiliary has a fixed wavelength at 1040 nm and pulses of about 140 fs. Both outputs have high quality beam with an average power of up to 1.5 W. The principal output is sent to a delay line, which can be adjusted in order to synchronize the pulses of both beams on the focal plane, a condition that varies depending on the wavelength set for the tunable laser, on the optics subsequently posed on the optical path, and on the depth of recording within the specimen. Then, the light of each beam passes through a mechanical shutter in order to minimize the exposure of the sample to the laser light, a telescope to regulate beam dimension and collimation, a motorized half-waveplate (λ/2) and a Glan-Taylor polarizer (PBS) for remote power adjustments and two mirrors to superimpose the beams on a dichroic mirror, which can be easily replaced (through a kinematic support) according to the wavelength chosen for the excitation. In order to overlap the two beams, the mirrors on the 1040 nm laser beam path are equipped with piezoelectric handlers. More in detail, after the dichroic the beams are sampled and split by two beamsplitters (10/90 and 50/50, respectively) and light signals are collected by two quadrant detectors, placed at different distances, in order to monitor beams superposition. We verified that such a condition provides a sufficient spatial overlap to generate the CARS signal at the focal volume. However, beam superimposition on the sample was further adjusted by closely looking at the image contrast achieved. After such optical elements, both beams are sent to the scanning system, placed on a vertical breadboard, for multi-photon imaging. Alternatively, a flipping mirror can be placed immediately before the breadboard in order to perform Raman spectroscopy. For this application, the laser source is a Toptica XTRA II (Toptica Photonics AG, Graefelfing, Germany) narrowband laser with 785 nm emission; as for the other optical paths, we placed a shutter and a telescope before the flipping mirror.

Upon the vertical breadboard, two galvanometric mirrors (Cambridge Technology, Bedford, MA, USA) and related scan and tube lenses respectively provide fast beam scanning and optical relaying with the objective lens. The excitation light passes through a kinematic support system and reaches a λ/2 waveplate for varying beam polarization in order to perform polarized-SHG (P-SHG) and polarized-CARS (P-CARS) measurements. For the first process, the polarized optics (QWP, HWP3) ensure a linear polarization at this point of the system, so that the waveplate can rotate it on the sample. For P-CARS, it is possible to adjust both beams independently, so the configuration that was found to be more efficient and stable has been experimentally identified, which provides the pump beam with circular polarization and the Stokes beam with linear polarization. In this way, the interposed waveplate has the effect of changing the sign of the polarization of the first beam, leaving it circular regardless of its position; instead, the polarization of the second beam is rotated analogously to the P-SHG case. This condition is obtained with the polarized optics placed close to the auxiliary mirrors for the superimposition (Figure 1). Finally, the objective focuses each laser on the sample. To realize 3D imaging, the objective is mounted on a support equipped with mechanical and piezoelectric (P-725KHDS PIFOC, Physik Instrumente, Karlsruhe, Germany) translators for gross and fine displacement, respectively. The samples are housed on an xy-translator (M-687 PIline, Physik Instrumente, Karlsruhe, Germany) which is able to map large areas, the acquired images in fact can be stitched together by attaching them, one beside the other.

Moreover, using a second water immersion objective Fluor 40× (Nikon, Tokyo, Japan), NA 0.8, WD 2 mm, it is possible to acquire forward-emitted signals. This optical access is also used to trans-illuminate the sample with wide-field, white light; a second flipping mirror is placed between the scan and tube lenses for capturing the transmitted white light onto a CCD camera (Thorlabs Inc., Newton, NJ, USA). The whole system is mounted on an Anti-Vibration Table (Thorlabs Inc., Newton, NJ, USA) equipped with active-air system.

### 2.2. The Detection System

**Figure 2:**
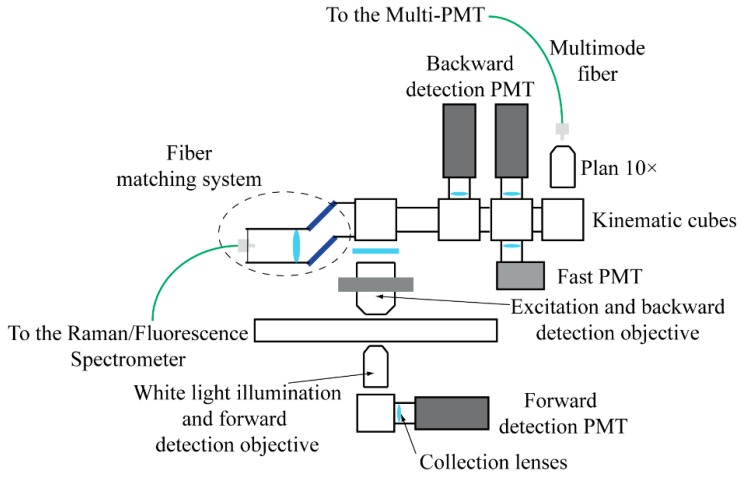
The detection system is composed by five kinematic cubes that hold filters and mirrors to send the light signals to the corresponding detectors. Spectral measurements are acquired by a spectrometer optically coupled through an optical fiber; alternatively, four PMTs enable epi and trans detection for multiphoton imaging and FLIM, while another multimode fiber transmits the collected light to a multispectral detector for SLIM measurements.

Our microscope is capable of recording images and spectra obtained through different optical techniques: Raman, TPEF, SHG, CARS and FLIM. Switching from one imaging/spectroscopic modality to the other simply requires a different arrangement of the optics mounted on kinematic supports (Figure 2).

**Figure 3:**
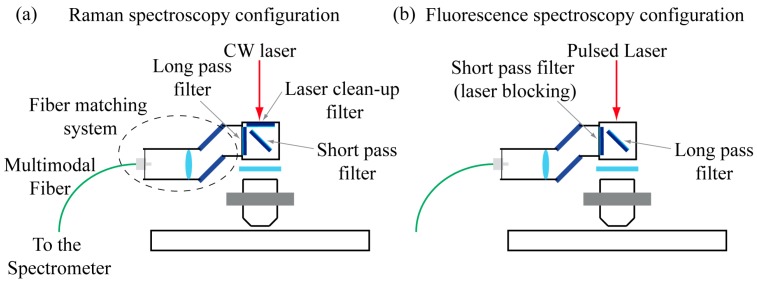
(**a**) The setup configuration used for Raman spectroscopy acquisitions, the signal is backward collected, separated from the excitation laser by a dichroic, coupled to an optical fiber and then delivered to the spectrograph. (**b**) The two-photon fluorescence spectroscopy configuration; in this case the laser light has longer wavelength and the filters have to be switched.

Raman spectroscopy measurements are performed by placing a short-pass dichroic filter (DMSP805R, Thorlabs Inc., Newton, NJ, USA) in the kinematic support above the excitation objective (Figure 3). The backward-collected signal is coupled into an optical fiber and delivered to the Kymera 328i imaging spectrograph (Andor, Belfast, UK), where an 830 grooves/mm diffraction grating resolve the spectrum. To optimize the measurement condition two optical filters are inserted: a narrow band-pass filter (FF01-780/12-25, Semrock Inc., New York, NY, USA) to clean-up the laser line and a notch filter (NF03-785E-25, Semrock Inc., New York, NY, USA) to prevent reflection of the laser light from entering the fiber. The spectrograph also makes it possible to spectrally resolve the two-photon fluorescence by: removing the band-pass filter, replacing the short-pass filter with a long-pass one (FF665-Di02-25x36, Semrock Inc., New York, NY, USA), replacing the notch filter with a high optical density short-pass one (F01-680/SP-25, Semrock Inc., New York, NY, USA), and using a different grating with 500 grooves/mm. The spectrograph was calibrated using a Neon-Argon glow lamp.

**Figure 4:**
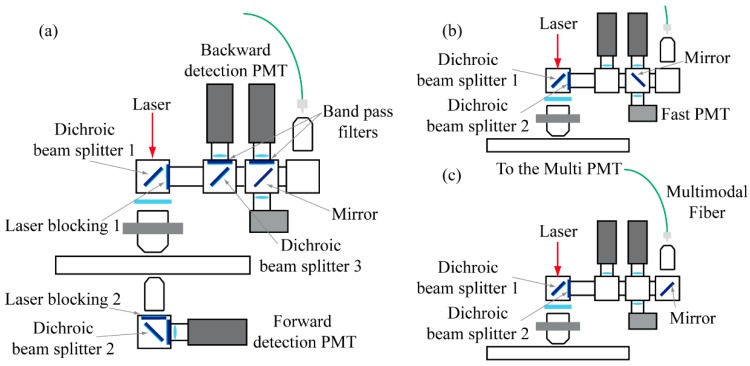
(**a**) Setup configuration used for MPM acquisitions, the signal can be collected both backward and forward. A system of filters makes it possible to separate the signal from the excitation and to detect each contrast mechanism onto a different PMT. (**b**) The setup configuration used for FLIM acquisitions. (**c**) The setup used for SLIM acquisition.

On the other hand, a typical configuration for MPM measurements consists of setting the tunable wavelength to around 800 nm and using it in tandem with the 1040 nm beam. This configuration makes it possible to generate SHG emissions at 400 or 520 nm, respectively, and TPEF emissions, whose spectrum starts at longer wavelengths than each SHG signal, ending around 650 nm. Using both sources at the same time, we can induce the CARS signal. Matching the C-H vibrational transition around 3000 cm^−1^, for example, results in a CARS emission centered about 640 nm. Figure 4a shows the filter configuration used for such measurement.

For backward-detection, in order to separate excitation and emission radiations, it is sufficient to turn the long-pass dichroic filter used for the fluorescence spectrum acquisition in the first cube of the kinematic support. Again, the high optical density short-pass filter prevents the laser light to reach the detectors. By means of other dichroic and optical filters, it is possible to separate SHG, TPEF and CARS signals and send them to two photomultiplier tubes H7422-40 (Hamamatsu, Hamamatsu City, Japan) through their relative collection lenses, thereby making is possible to acquire simultaneously different MPM signals via photocurrent integration. For forward-detection, another high optical density short-pass filter attenuates the laser light and a long-pass filter sends the excited signal from the sample toward a third PMT. In particular, SHG and CARS signals were selected by narrow band-pass filters, while a large band-pass filter was used for collecting TPEF emission.

As shown in Figure 4b, the same kinematic system can be used to send the light to a PMH-100 detector (Becker-Hickl GmbH, Berlin, Germany) to perform FLIM measurements, or (Figure 4c) to an objective lens Plan 10× (Nikon, Tokyo, Japan) that focuses the light into a multimode optical fiber, connected to a multispectral detector (Multi-PMT) for SLIM microscopy. In particular, the PML-Spec Multi-PMT (Becker-Hickl GmbH, Berlin, Germany) is composed by a diffraction grating with 600 lines/mm and a 16-channels multi-anode photomultiplier strip with 200 ps FWHM pulse width.

### 2.3. The Control and Acquisition System

A custom-made software developed in LabVIEW 2015 (National Instruments, Austin, TX, USA), allowed us to control the motorized stages, to set and program the features of the laser beams and the relative position between objective and sample, to start excitation and acquisition by triggering two synchronized I/O boards, and to build the images from the acquired signals. The I/O boards are a PCI-MIO (National Instruments, Austin, TX, USA) and a SPC-730 (Becker-Hickl GmbH, Berlin, Germany). The former board controls the movements of shutters, scanning mirrors and acquires the amplified and integrated signals of the PMTs; the latter board is dedicated to time-resolved single photon counting measurements. In this case, the visualization of the acquired images is accomplished using the dedicated software, SPCM 9.81 (Becker-Hickl GmbH, Berlin, Germany), that also allowed us to change the photon counting board settings. Image pixels exponential fits, de-convolution and fluorescence decay analyses are performed using the software SPC-Image 2.8 (Becker-Hickl GmbH, Berlin, Germany). Raman and fluorescence spectrum acquisitions were performed using the software Andor Solis (Andor, Belfast, UK). Images were handled with ImageJ and graphs and histograms were prepared in Microcal Origin Pro 8.1 (OriginLab Corporation, Northampton, MA, USA).

### 2.4. Sample Description and Preparation

To test the microscope, we prepared a solution containing gold nanoparticles (50 nm size) through a seed-mediated synthesis and imaged them for evaluating its spatial resolution. Then, we used human tissue samples for showing the functioning and potential applications of its imaging and spectroscopic techniques.

Notably, we examined biopsies of healthy bladder mucosa, urothelial carcinoma (UC) and atherosclerotic carotid. Four fresh biopsies of urothelial tumor and healthy bladder mucosa, collected from patients undergoing TURBT, were frozen in liquid nitrogen and then stored at −80 °C. Two “bulk” biopsies were fixed between a slide and a coverslip - both made of quartz in order to avoid spurious Raman contributions from glass - while the other two were sliced with a microtome, without paraffin embedding; the resulting 30-µm-thick slices were placed on a different microscope slide. One human atherosclerotic carotid specimen was excised through surgery, fixed in formalin and embedded in paraffin. Two adjacent cross-section slices were obtained: a 3-µm-thick slice stained with H&E, and a 30-µm-thick slice unstained and placed on a slide for MPM imaging. This allowed us to perform a direct comparison of the MPM images with the corresponding histological images. Both bulk biopsies and thin tissue slices were examined with our multimodal microscope. All measurements involving human samples that are reported in this study were approved by the local Ethical Committee and conducted according to the tenets of the Declaration of Helsinki, after having obtained an informed consent from all the subject participating in the study.

## 3. Results and Discussion

### 3.1. Microscope Calibration: Spatial and Temporal Resolution

**Figure 5:**
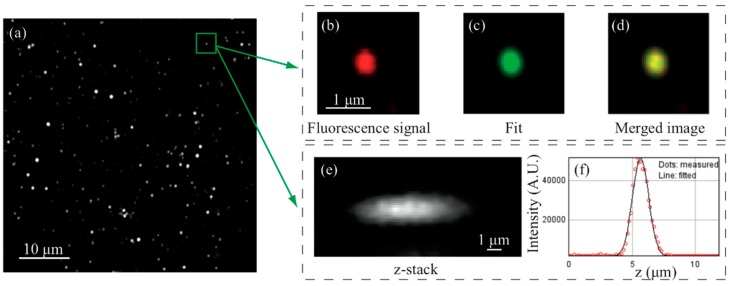
(**a**) Fluorescence signal from the gold nanoparticles, (**b**) one individual gold nanoparticle in red, (**c**) 2D Gaussian fit and (**d**) merged image. (**e**) z-stack reconstruction and (**f**) relative Gaussian fit. The measurement was performed under the following experimental conditions: water immersion (n = 1.33), 800 nm excitation wavelength, 0.95 objective numerical aperture (NA), 100 μm × 100 μm FOV, 2048 pxl × 2048 pxl resolution, and with 200 nm axial steps.

In two-photon microscopy, the spatial resolution corresponds to the dimensions of the excitation volume which is smaller than the beam waist because of non-linear properties of excitation. In order to measure this parameter in our optical system, we considered the point spread function (PSF) as the measures size of a sub-diffraction limit object, such as water-diluted gold nanoparticles with an average diameter of 15 nm. The optical scanning in the three spatial directions was accomplished using water (n = 1.33) as an immersion medium in a large field with many nanoparticles, and by performing a statistical analysis using the PSFj software [29]. The distance between two adjacent pixels in X and Y directions was calculated after calibration performed moving the xy-stage. Figure 5a shows an image of the fluorescence signal emitted by the nanoparticles, together with a magnified detail of an individual nanoparticle (Figure 5b), the corresponding radial fit (Figure 5c) and the merged image (Figure 5d). Acquiring the fluorescence signal changing the focal plane with steps of 200 nm, is possible to build the axial image (Figure 5e) and perform the fit (Figure 5f). The spatial resolution was then evaluated as the full width half maximum (FWHM) of the fitting functions, a 2D Gaussian in the radial plane and a 1D Gaussian along the optical axis. The averaged values are 396 ± 5 nm for the minimum radial FWHM, 467 ± 7 nm for the maximum radial FWHM and 1.64 ± 0.05 μm for the axial FWHM. The obtained results are comparable with the theoretical values of 235 nm and 1.04 um for radial and axial resolutions respectively, that are derived from the formulas reported in [4].

**Figure 6:**
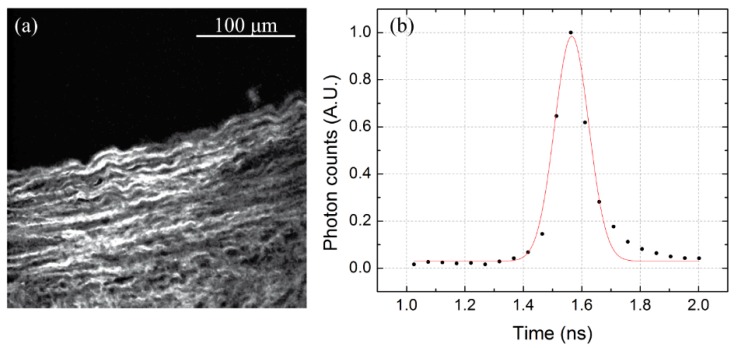
(**a**) SHG image of a collagen sample. (**b**) The corresponding time-resolved signal with a superimposed Gaussian fit.

The time resolution of a Time Correlated Single Photon Counting (TCSPC) system is described by the total instrument response function (IRF), which is the convolution product between the impulse response function of every element of the system. In order to measure the IRF of the system, we have analyzed the time response to a laser pulse. Considering that the FWHM of the laser pulse (100 fs) is about three orders of magnitude smaller than the expected FWHM of the IRF, it is then possible to approximate the laser pulse as a δ-function in time and to use it to obtain the total IRF of the system. In order to avoid potential damaging of the detectors due to the high intensity power of a laser pulse, we chose to detect a back-reflected SHG from a biological sample. The SHG process is a coherent, instantaneous, phenomenon which provides an adequate δ-function signal with adequate intensity to avoid potential damage to the detectors. More in detail, the SHG signal from a sample of collagen fibers has been acquired with the PMH-100, exciting with the tunable laser at 840 nm and by placing a band-pass filter (FF01-420/10-25, Semrock Inc., New York, NY, USA) in front of the detector. The time-resolved signal is plotted in Figure 6 and fitted to a Gaussian function. The observed FWHM was approximately (150 ± 50) ps, where the uncertainty is given by the temporal binning of the measurement (12500 ps/256 time channels). Such a result is consistent with the IRF of the PMH-100 detector provided by the producer, whose datasheet certifies it as 150 ps. Therefore, the obtained IRF will be used in the following fluorescence lifetime measurements to deconvolve the acquired decays and eliminate the contribution due to the instrumental response.

### 3.2. Multimodal Imaging of Human Ex Vivo Tissue Samples

Our multimodal microscope is capable of examining bulk biopsies as well as thin tissue slices. On one hand, the optical inspection of a bulk biopsy could contribute to reducing the time needed for tissue fixation, embedding and slicing procedures. On the other, imaging tissue slices makes it possible to make direct comparisons with standard histopathological results. Moreover, mapping large tissue areas can improve the assessment of disease progression (for example, by monitoring the size of necrotic areas or the degree of tumor infiltration). In order to prove its potential in various kinds of specimens, we tested our instrument on both bulk biopsies and thin tissue slices, obtained from tumor and non-tumor diseases.

**Figure 7:**
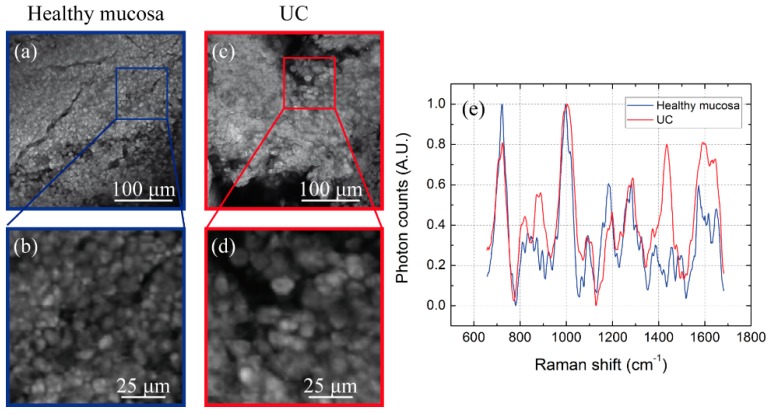
(**a**) TPEF images of healthy mucosa (blue) with (**b**) magnified detail, (**c**) UC tissues (red) with (**d**) magnified detail, (**e**) average Raman spectra collected in the magnified regions.

We firstly examined two bulk biopsies of human bladder: one excised from healthy bladder, the other one from UC tissue. UC accounts for about 90% of all bladder tumors, and is one of the most common types of cancer [30]. Presence, invasiveness and grade of UC are generally diagnosed by histopathological examination of several tissue biopsies obtained through Transurethral Resection of Bladder Tumor (TURBT). A key indicator of malignancy, for example, is the dimension of cell nuclei, which is significantly bigger in UC with respect to normal bladder [31]. These samples are generally difficult to diagnose, even for an experienced pathologist, since the specimens are small and soft, making proper orientation for slicing not so trivial. In this framework, combining MPM and Raman spectroscopy could be a valuable alternative for high-resolution imaging and diagnostics of bladder tissues. Figure 7 shows the TPEF images recorded from healthy mucosa (blue) and UC (red) biopsies. Fluorescence imaging takes advantage of intrinsic fluorescent molecules (contained both in cells and in the extracellular matrix) such as NADH, tryptophan, keratins, elastin and others. Hence TPEF can be used to evaluate the different morphology of the examined tissues: in fact, from these images we can observe the different size between normal and tumor cells, that have high fluorescent cytoplasm. After imaging normal and tumor areas through MPM, we used Raman spectroscopy to obtain additional information on the molecular content of these tissues from specific areas of interest. In particular, we collected two Raman spectra by scanning the 785 nm laser along a squared portion of 100 μm size from each TPEF image; this approach made it possible to probe the molecular content of large tissue areas within few seconds, instead of performing a very long Raman mapping. The results are shown in Figure 7: the Raman spectra collected from healthy mucosa (blue, 7b) and from UC (red, 7d) regions show significant differences between their spectral profiles. In particular, UC area shows two main peaks around 900 and 1400–1500 cm^−1^, whereas healthy bladder region does not. The first band is typically associated with amino acids (such as proline [32]), while the second corresponds to proteins (such as collagen) and lipids [33]. These differences suggest that Raman spectroscopy could be used to probe the composition of specific areas in tissue biopsies and, potentially, to highlight the altered metabolism [34] of bladder cancer with respect to normal mucosa.

**Figure 8:**
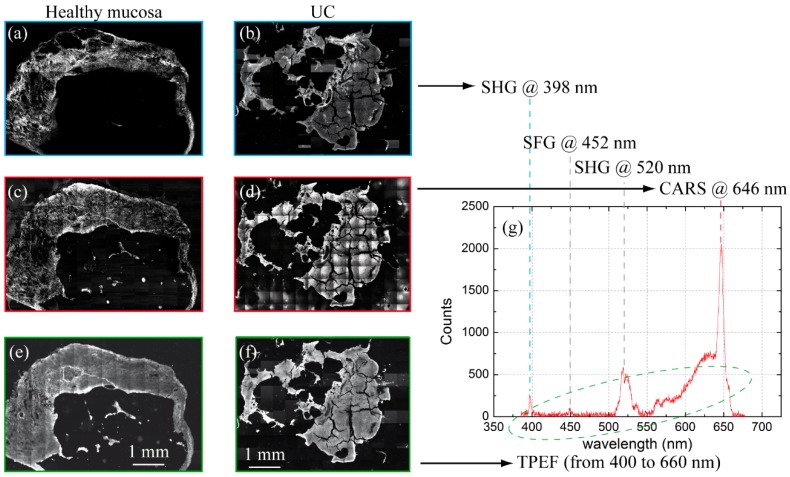
(**a**–**b**) SHG images acquired with the 797 nm pulsed laser from healthy mucosa and UC respectively, (**c**–**d**) CARS mapping using both lasers, (**e**–**f**) TPEF images acquired with 797 nm pulsed laser. (**g**) Multi-photon spectrum, excited with synchronized pulsed lasers emitting at 797 nm and 1040 nm, presenting two narrow SHG signals at 398 and 1040 nm respectively, a peak at 452 nm due to the Sum Frequency Generation (SFG) process, CARS peak at 646 nm and the fluorescence which is broad and covers the 400-650 nm range. The spectrum is very attenuated in the first half due to the optics, which are optimized for the Raman signals in the near infrared, while the cut around 665 nm is due to the dichroic for the signal collection.

Then, we used MPM to image tissue slices obtained from other two biopsies (one from healthy bladder mucosa, one from UC). The sliced tissue samples allowed us to map each specimen, as shown in Figure 8. Together with TPEF, we acquired also SHG and CARS signals in order to highlight the presence of, respectively, collagen structures and CH_2_ bonds, which are particularly abundant in lipid accumulations and characterized by a strong Raman band around 2900–2940 cm^−1^ [35,36]. As explained in Section 2.2, we used different setup configurations and optical filters to separate SHG, TPEF and CARS signals, whose spectral emissions are clearly visible in the reported spectrum.

Then, we examined paraffin-embedded atherosclerotic slices in order to evaluate the potential of combined SHG-TPEF-FLIM microscopy for tissue characterization. Atherosclerosis is a non-tumor disease consisting of slow, progressive accumulation of cholesterol, fatty substances, cellular waste products, macrophages, calcium and fibrin in the artery wall, which can generate plaques [37,38,39]. The sudden rupture of a vulnerable plaque delivers thrombotic material within blood, potentially resulting in a heart attack or a stroke. In this regard, plaque stability is highly correlated to its composition [40]: stable plaques are rich in smooth muscle cells and show a thick fibrous cap, while unstable ones are characterized by large necrotic cores, calcification, few smooth muscle cells and a thin cap. Hence, atherosclerosis is an interesting case-study for MPM: these techniques could be used [26,41] to examine the plaque composition and to distinguish between different atherosclerotic tissues, resulting in a faster and label-free alternative to standard histology for the assessment of plaque severity. Since the fibrous cap is rich in collagen fibers, SHG can provide specific contrast from the rest of the plaque—which, instead, is rich in endogenous fluorophores that can be imaged through TPEF microscopy. An analysis based on fluorescence intensity and lifetime could also help in discriminating other atherosclerotic tissues due to their different morphology and molecular content.

**Figure 9:**
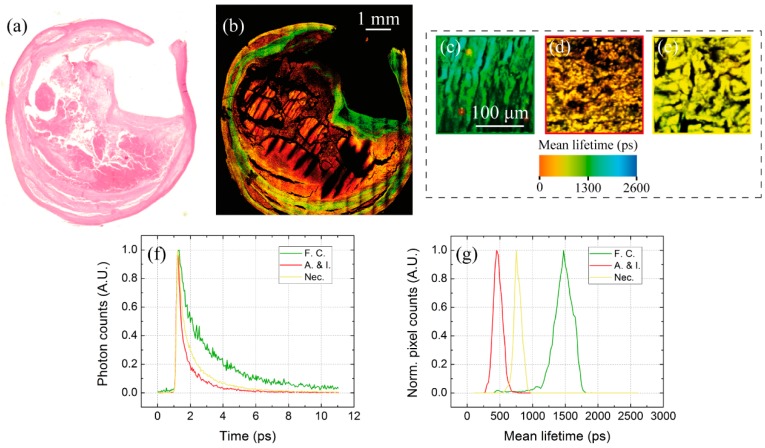
(**a**–**b**) H&E image of a human carotid cross-section and MPM false color mapping (SHG green, TPEF red) of the adjacent unlabeled slice. (**c**–**e**) False color FLIM images of fibrous cap (F.C.), angiogenesis and inflammation (A.& I.) and necrotic (Nec.) tissues. (**f**) Fluorescence decays recorded from the three tissues, with the distributions (**g**) of their corresponding mean lifetime values.

Figure 9 shows the merge of SHG and TPEF mappings of a large carotid specimen. As expected, the collagen-rich fibrous cap areas around the arterial lumen can be easily detected by SHG with significantly higher contrast with respect to the corresponding H&E image; this result is particularly important for measuring fibrous cap thickness and, thus, estimating plaque vulnerability. Another relevant factor is the presence of necrotic and inflamed areas, whose different morphology (Figure 9d,e) can be observed though TPEF microscopy. These tissues are also characterized by strong differences in their molecular content, but TPEF lacks the capability to discriminate them due to the overlapping absorption spectra of typical endogenous fluorophores. In this regard, the superior specificity of FLIM enables the identification of fibrous cap (F.C), necrotic (Nec.) and angiogenesis/inflamed (A.&I.) areas through the analysis of their fluorescence decays (Figure 9c–g).

**Figure 10:**
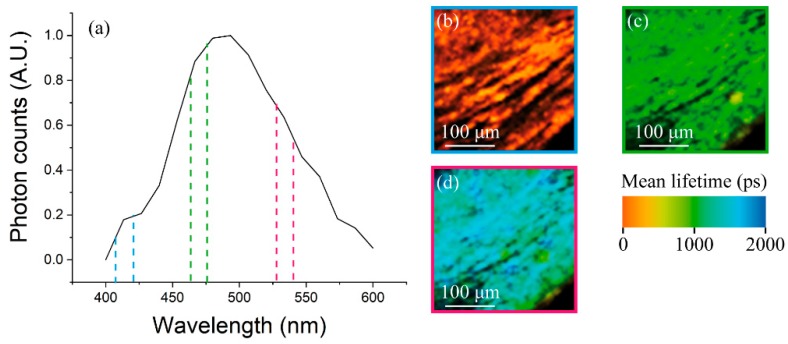
(**a**) The spectrum acquired with the 16 channels of the PML-Spec Multi-PMT, (**b**–**d**) the false colors FLIM images acquired in the second, sixth and eleventh channels, which correspond to SHG emission from collagen fibers, TPEF from elastin and collagen, and TPEF from smooth muscle cells, respectively.

While the same wavelength can excite several fluorophore types, their emission peaks can vary significantly. Thus, spectral lifetime analysis provides an additional characterization of biological tissues: SLIM makes it possible to separate the contributions of different fluorescent emitters, and then analyzes their time decay. This feature could help in identifying the molecular content of atherosclerotic tissues. For example, we tested this imaging technique by examining a portion of the carotid wall with our 16-channels detector. Figure 10 shows the spectrum collected between 400 and 600 nm using the pulsed laser with 840 nm excitation: the first channel (≈400 nm) presents no signal, while the second one (≈420 nm) is characterized by a very fast decay due to SHG emission from collagen fibers. The remaining channels collected the fluorescence signal, with its characteristic emission peak around 500 nm and decay times of ≈1–2 ps. As found in a previous study [40], different fluorophores contribute to different spectral channels. Fluorescence from collagen and elastin fibers is peaked around 470 nm, while smooth muscle cells emit mainly between 500 and 550 nm. Figure 10b–d report the FLIM images acquired from three specific channels, which in fact reflect different lifetime values due to—respectively—collagen SH emission, elastin and collagen fluorescence, and smooth muscle cells fluorescence emission. The significant lifetime differences observed between the three images demonstrate the capability of SLIM to probe tissue composition and molecular environment, which are relevant factors in assessing plaque vulnerability.

## 4. Conclusions

In this paper, we presented a custom-made, multiple-light-source, laser-scanning microscope that (to our knowledge) is unique. The combination of SHG, TPEF, CARS, Raman, FLIM, and SLIM in the same experimental setup represented not only the overcoming of a technical challenge, but also a significant improvement in the characterization of biological tissues. In fact, the capabilities of this multimodal approach are far beyond those of each single technique, or even of their combined use though multiple setups (which would be a much slower and more complicated solution). Using a single instrument for all imaging/spectroscopic techniques makes it possible to obtain an unparalleled level of tissue information at the micron-scale. In this regard, the calibration of our system proved its sub-cellular spatial resolution and sub-ns temporal resolution, while the tests performed on human ex vivo tissues confirmed the successful application of MPM, FLIM and Raman spectroscopy to the study of both tumor and non-tumor diseases. In order to perform these measurements, we arranged multiphoton imaging and optical spectroscopy in a unique and complementary approach: the MPM for studying tissue morphology and detecting (in a relatively short amount of time) specific regions of interest, then scanning each region, allowing us to record its average Raman spectrum or fluorescence decay, thus providing detailed information about its molecular composition. In this way, the speed of multiphoton imaging was integrated with the specificity of Raman and FLIM spectroscopy, avoiding the extremely time-consuming alternative of a Raman or FLIM mapping over a broad area. The presented results and discussion suggest that our microscope could be used to study a wide range of biomedical problems in a fast and label-free modality, providing an additional tool for, and potential alternative to, standard histopathological examination.
